# Heavy Metal, Heavy Heart: Adverse Cardiovascular Effects of Uranium Exposure

**DOI:** 10.1016/j.jacadv.2024.101404

**Published:** 2024-11-18

**Authors:** Omar Hahad, Sadeer Al-Kindi

**Affiliations:** a Department of Cardiology–Cardiology I, University Medical Center of the Johannes Gutenberg University Mainz, Mainz, Germany; b German Centre for Cardiovascular Research (DZHK), Partner Site Rhine-Main, Mainz, Germany; c Division of Cardiovascular Prevention and Wellness, Houston Methodist DeBakey Heart and Vascular Center, Houston, Texas, United States; d Center for Health and Nature, Houston, Texas, United States

**Keywords:** cardiovascular disease, heavy metals, pollution

**Figure:**
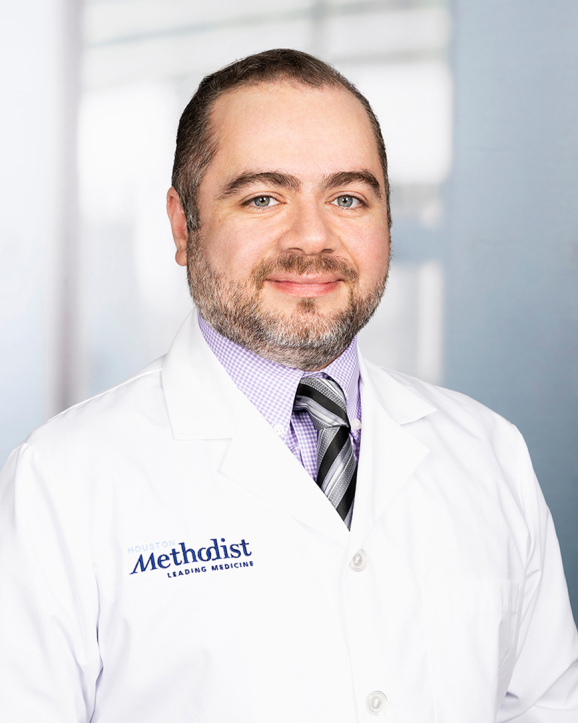

The cardiovascular effects of heavy metal exposure are well-documented.^1^^,^^2^ Historical reports, dating back over a century, highlighted a rise in hypertension and stroke cases among workers exposed to lead.^1^ Since then, extensive epidemiologic and experimental research has established a robust link between exposure to various (heavy) metals—such as arsenic, lead, cadmium, mercury, and copper—and adverse cardiovascular disease outcomes, highlighting their cardiotoxic effects.^1^^,^^3^

Importantly, uranium contamination of soil and water represents a global health concern that remains insufficiently studied, with significant regional variation in uranium concentrations due to both natural (geogenic) and human-made (anthropogenic) sources. In the United States, this contamination is prevalent, with over half of public water systems showing detectable levels of uranium. Although food and air can also be sources of exposure, drinking water remains the primary route for most people. Certain communities, particularly American and Indian populations, are at greater risk due to their reliance on contaminated water sources and their proximity to uranium mining and milling activities.^4^^,^^5^

The health effects of long-term, low-level uranium exposure are not yet well-understood. Although the nephrotoxicity of uranium is well-established,^6^ emerging data suggest it may also affect cardiovascular health. Animal studies have shown that uranium exposure can lead to oxidative stress, inflammation, and DNA damage in cardiac tissues.^7^ Given the widespread presence of uranium in drinking water and its potential cardiovascular toxicity, there is a critical need to evaluate the long-term cardiovascular effects of low-level uranium exposure in human populations.

In this issue of *JACC: Advances*, Lieberman-Cribbin et al^8^ aim to fill this research gap by exploring the relationship between urinary uranium concentrations and measures of cardiac structure and function in young adults from American Indian communities, as part of the Strong Heart Family Study. This cohort included 1,332 participants, all free from diabetes and cardiovascular disease, and under the age of 50 years at baseline (2001-2003). Urinary uranium levels were measured at the start of the study, while echocardiograms and blood pressure assessments were conducted both at baseline and during follow-up between 2006 and 2009. The researchers employed linear mixed effects models to estimate the adjusted mean differences in cardiac measures in relation to urinary uranium concentrations.

The median urinary uranium concentration in this cohort was 0.029 μg/g creatinine, higher than levels observed in the general U.S. population.^7^ Cross-sectional analyses indicated that a doubling of urinary uranium levels was associated with increased left ventricular mass index, left atrial systolic diameter, and stroke volume at baseline, suggesting that even at relatively low concentrations, uranium exposure may induce subtle changes in cardiac structure. Longitudinal analyses further supported this, revealing associations between uranium exposure and increases in left atrial diameter, pulse pressure, and the development of left ventricular hypertrophy over the follow-up period. Notably, the observed increase in pulse pressure is of special importance, as it is a known risk factor for cardiovascular events and could be indicative of heightened arterial stiffness.^9^, ^10^, ^11^

This study presents compelling evidence that even low-level uranium exposure may negatively impact cardiac structure and function in young, otherwise healthy adults. The observed changes, including left ventricular hypertrophy and left atrial enlargement, are well-established predictors of future cardiovascular events.^12^^,^^13^ The magnitude of these effects, though modest, is similar to those seen with traditional cardiovascular risk factors, suggesting that uranium exposure may represent a significant public health issue (Figure 1).

**Figure 1:**
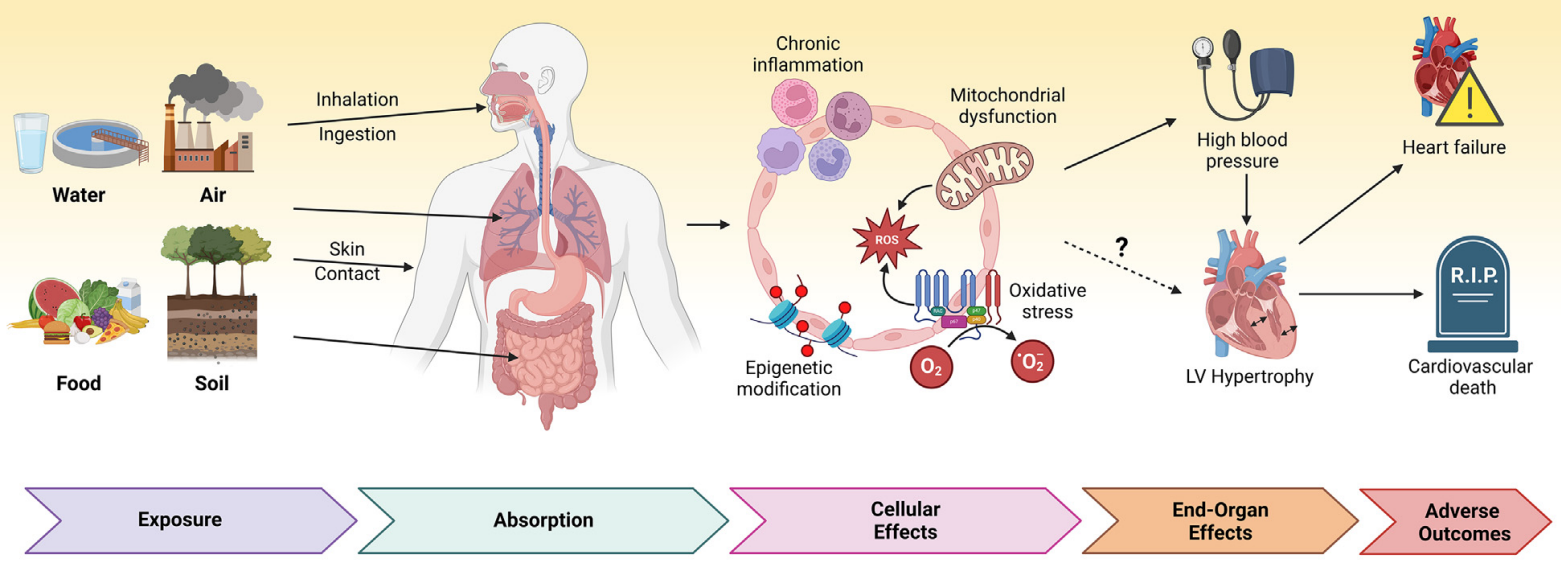
Pathways Linking Uranium Exposure to Left Ventricular Hypertrophy: From Environmental Exposure to End-Organ Damage

The strengths of this study include its prospective design, comprehensive evaluation of cardiac structure and function, and the use of urinary uranium as a biomarker of chronic exposure. Additionally, the authors adjusted for a variety of potential confounding variables, including co-exposures to other metals and socioeconomic factors, which enhance the robustness of the findings. However, limitations should be acknowledged. A single urine measurement may not fully capture long-term exposure patterns and variations, and, as with all observational studies, residual confounding cannot be entirely excluded. Furthermore, the focus on American Indian populations may limit the generalizability of the findings to other groups with different exposure levels or genetic backgrounds. Additionally, echocardiographic measurements of left ventricular mass have limitations, including potential variability in technique and operator dependency, which may introduce measurement error.

Despite these limitations, this research has significant implications for public health and clinical practice. It underscores the need for further studies into the mechanisms by which uranium impacts cardiovascular health, including its role in oxidative stress, inflammation, and epigenetic changes. These findings also highlight the importance of incorporating environmental uranium exposure into cardiovascular risk assessments, especially in areas with known contamination. The results raise also important issues related to environmental justice and health equity. Disproportionate exposure to uranium and other environmental risk factors in certain communities may contribute to ongoing health disparities. Addressing these disparities requires targeted research and policy efforts to ensure all populations have access to clean water and safe environments. From a public health perspective, these results call for increased efforts to monitor and mitigate uranium contamination in drinking water, particularly in vulnerable communities.^14^ Clinicians, particularly those working in regions with known uranium contamination, should be aware of the potential cardiovascular risks when treating patients from these areas.

Future research should aim to replicate these findings in more diverse and larger populations, investigate dose-response relationships, and explore potential interventions to counter the cardiovascular effects of uranium exposure. Additionally, studying the combined effects of uranium and other environmental exposures, such as air pollution or heavy metals, may provide valuable insights into the complex interactions of environmental risk factors in the development of cardiovascular diseases.

## Funding support and author disclosures

Dr Al-Kindi is funded by the 10.13039/100009720Jerold B Katz Foundation. Dr Hahad has reported that he has no relationships relevant to the contents of this paper to disclose.
